# Osteoblast differentiation of equine induced pluripotent stem cells

**DOI:** 10.1242/bio.033514

**Published:** 2018-04-23

**Authors:** Arabella Baird, Timothy Lindsay, Alice Everett, Valentine Iyemere, Yasmin Z. Paterson, Alyce McClellan, Frances M. D. Henson, Deborah J. Guest

**Affiliations:** 1 Centre for Preventive Medicine, Animal Health Trust, Lanwades Park, Kentford, Newmarket, Suffolk, CB8 7UU, UK; 2 Division of Trauma and Orthopaedic Surgery, University of Cambridge, Addenbrooke's Hospital, Cambridge, CB2 0QQ, UK; 3 Department of Veterinary Medicine, University of Cambridge, Madingley Road, Cambridge, CB3 0ES, UK

**Keywords:** Equine, Induced pluripotent stem cells, Osteoblast, Differentiation, Gene expression

## Abstract

Bone fractures occur in horses following traumatic and non-traumatic (bone overloading) events. They can be difficult to treat due to the need for the horse to bear weight on all legs during the healing period. Regenerative medicine to improve fracture union and recovery could significantly improve horse welfare. Equine induced pluripotent stem cells (iPSCs) have previously been derived. Here we show that equine iPSCs cultured for 21 days in osteogenic induction media on an OsteoAssay surface upregulate the expression of osteoblast associated genes and proteins, including *COL1A1*, *SPARC*, *SPP1*, *IBSP*, *RUNX2* and *BGALP*. We also demonstrate that iPSC-osteoblasts are able to produce a mineralised matrix with both calcium and hydroxyapatite deposition. Alkaline phosphatase activity is also significantly increased during osteoblast differentiation. Although the genetic background of the iPSC donor animal affects the level of differentiation observed after 21 days of differentiation, less variation between lines of iPSCs derived from the same horse was observed. The successful, direct, differentiation of equine iPSCs into osteoblasts may provide a source of cells for future regenerative medicine strategies to improve fracture repair in horses undergoing surgery. iPSC-derived osteoblasts will also provide a potential tool to study equine bone development and disease.

## Abstract (precis)

**Summary:** Horse iPSCs can be turned directly into bone forming cells but the efficiency is affected by the donor horse. Less variability is observed using different iPSC lines derived from the same horse.

## INTRODUCTION

Fractures occur in horses from a direct trauma, such as a kick, as well as from bone overloading. They occur in all types of horses (Morgan and Dyson, 2012), with horses that take part in strenuous activities such as racing being at particularly high risk of having a fracture resulting from overloading (Verheyen and Wood, 2004). Numerous environmental risk factors have been identified that contribute to fracture risk (Anthenill et al., 2007; Kristoffersen et al., 2010; Parkin et al., 2004; Verheyen et al., 2006). More recently, fracture in racing Thoroughbred horses has also been shown to have a genetic basis (Blott et al., 2014; Welsh et al., 2014). Similarly, genetic (Bulathsinhala et al., 2017; Korvala et al., 2010; Zhao et al., 2016) and environmental (Warden et al., 2006, 2007) factors have also been implicated in stress fractures in human athletes and military personnel.

While severe fracture in horses usually leads to euthanasia, smaller fractures can be treated conservatively with box rest and a cast. In delayed union or comminuted fractures, surgery is required (Cahn, 2010), but up to 40% of horses do not return to their previous athletic activity (Payne and Compston, 2012). Regenerative medicine to improve fracture reunion and recovery could therefore significantly improve horse welfare (Govoni, 2015). Bone grafts are used to promote bone regeneration and restore normal bone architecture in humans, however, it is difficult to obtain sufficient tissue without donor site morbidity. Using stem cells to enhance tissue healing is therefore becoming a popular alternative (Ma et al., 2014).

Equine mesenchymal stem cells (MSCs) are widely reported to differentiate into bone (De Schauwer et al., 2011; Glynn et al., 2013; Guest et al., 2008; Nino-Fong et al., 2013). However, like human MSCs, equine MSCs are heterogeneous and show donor variability in many of their properties including osteoblast differentiation (Carter-Arnold et al., 2014). Methods have been established to differentiate human induced pluripotent stem cells (iPSCs) into osteoblasts with the aim of using these cells in bone tissue engineering strategies and modelling human bone disorders, reviewed in (Csobonyeiova et al., 2017).

Equine induced pluripotent stem cells (iPSCs) have been derived by ourselves (Bavin et al., 2015) and others (Breton et al., 2013; Khodadadi et al., 2012; Lee et al., 2016; Nagy et al., 2011; Quattrocelli et al., 2016; Sharma et al., 2014; Whitworth et al., 2014). Their ability to undergo differentiation into the three germ layers and capacity for unlimited self-renewal *in vitro*, underpins their potential applications in regenerative medicine. Establishing methods to differentiate iPSCs into bone forming osteoblasts would therefore open up the possibility of using these cells clinically to improve fracture repair after surgery or in *in vitro* disease modelling of bone disorders. However, due to the pluripotent nature of iPSCs, robust methods for differentiation need to be established to ensure that no undifferentiated iPSCs with the potential to form tumours remain (Liu et al., 2013).

Horses can provide a useful large animal model for human musculoskeletal injuries (Innes and Clegg, 2010). Unlike small laboratory species, horses naturally suffer from musculoskeletal conditions and therefore, horse repair models may more closely resemble the human situation. Added to this, the equine musculoskeletal system is of a more similar size and load bearing stress to that of humans. This allows the assessment of treatments for injuries with similar dimensions to those observed in human patients in the clinic. The horse therefore provides a relevant species with which to investigate the genetic mechanisms involved in fracture risk and to test novel regenerative therapies.

Here, we demonstrate for the first time that equine iPSCs can differentiate directly into functional osteoblasts *in vitro*.

## RESULTS

### Equine iPSCs derivation

Five lines of iPSCs were derived from three donor horses (one line from each of two horses, three lines from one horse). Two of the iPSC lines used in this study have previously been characterised for their normal karyotype, expression of pluripotency markers, and ability to differentiate into derivatives of endoderm, ectoderm and mesoderm (Bavin et al., 2015). The remaining lines underwent the same characterisation. All lines expressed pluripotency markers and underwent spontaneous differentiation into endoderm, ectoderm and mesoderm when cultured in the absence of feeder cells, leukaemia inhibitory factor, and basic fibroblast growth factor (Fig. S1).

### Equine iPSCs differentiate into cells with the molecular signature of osteoblasts

**Fig. 1.**
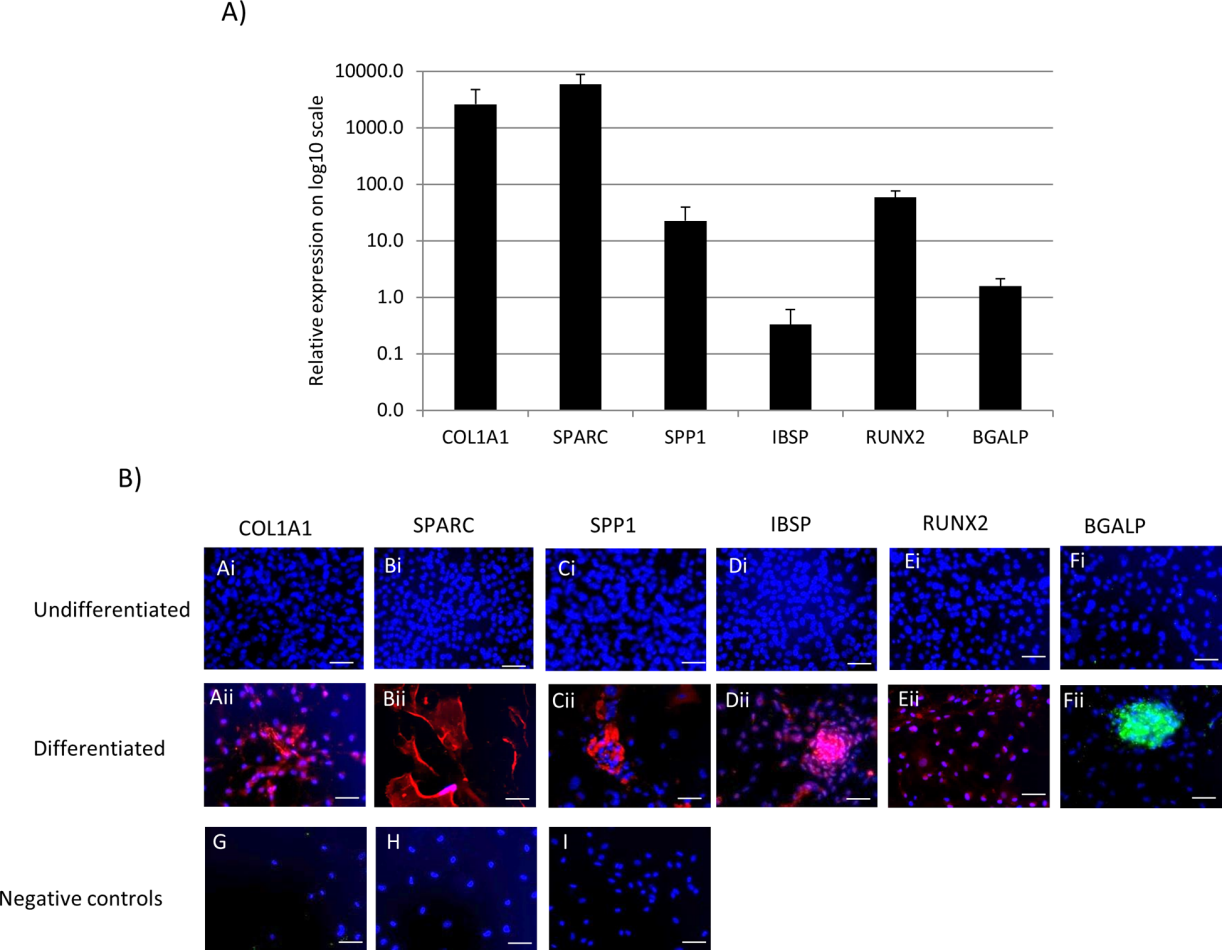
**Osteoblast-associated gene and protein expression** **in equine iPSCs differentiated in osteogenic media on an OsteoAssay surface for 21 days.** (A) Quantitative PCR analysis of osteoblast-associated genes. The mean of three independent replicates is shown. Error bars represent the s.e.m. and the relative expression (to the 18S housekeeping gene) is plotted on a log10 scale. (B) Immunocytochemistry is shown to detect osteoblast-associated proteins in undifferentiated iPSCs and iPSCs after 21 days of differentiation in osteogenic media. All proteins are detected following differentiation [(Ai–Eii) red staining; (Fi,Fii) green staining]. Negative controls for the secondary antibodies were performed on differentiated iPSCs (G) donkey anti-goat alexafluor 594. (H) Goat anti-rabbit alexafluor 594. (I) Goat anti-mouse FITC. DAPI staining of cell nuclei is shown in blue. Scale bars: 40 μm. Immunocytochemistry was performed on all lines of iPSCs and representative images are shown.

When cultured in osteogenic media on a surface designed to promote osteogenic differentiation [OsteoAssay (OA)], iPSCs differentiated into cells that expressed *COL1A1* (collagen type I), *SPARC* (osteonectin), *SPP1* (osteopontin), *IBSP* (bone sialoprotein), *RUNX2* (Runt related transcription factor 2) and *BGALP* (osteocalcin) (Fig. 1).

### Equine iPSCs produce osteoblast-associated proteins following differentiation

Undifferentiated iPSCs do not express any detectable osteoblast-associated proteins, whereas following 21 days in osteoblast induction media, COL1A1, SPARC, SPP1, IBSP, RUNX2 and BGALP are all detected in the differentiated cells (Fig. 1).

### Equine iPSC osteoblasts are functional and can produce a calcified mineralised matrix

**Fig. 2.**
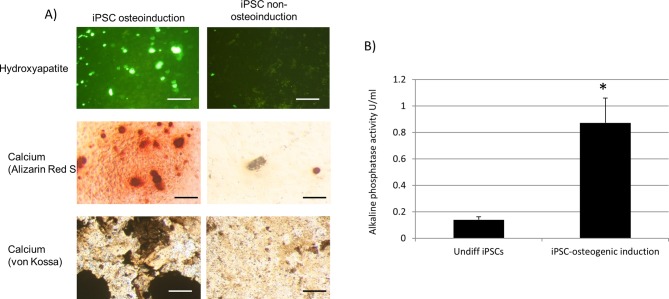
**iPSCs produce functional osteoblasts.** (A) A mineralised matrix is deposited by iPSCs cultured in osteoinduction media for 21 days but not by cells cultured in non-osteoinductive media for the same time period. Positive hydroxyapatite staining detected under fluorescent light is shown in green. Positive staining for calcium deposition is shown in red for Alizarin Red S and black for von Kossa. Scale bars: 100 μm. Representative images from across all lines of iPSCs are shown. (B) Alkaline phosphatase activity is significantly upregulated following 21 days of differentiation of iPSCs on an OsteoAssay surface in osteogenic media. An unpaired Student's *t*-test was used to demonstrate statistically significant differences in the mean ALP values (*P<0.05).

In the presence of osteogenic induction media, iPSCs stained positively for hydroxyapatite and calcium deposition after 21 days of culture. In contrast, there was very little, or no detection of hydroxyapatite and calcium deposition after 21 days when the cells were cultured in non-osteogenic induction media (Fig. 2). Hydroxyapatite was detected with a commercial bone mineralisation assay. Calcium deposition was detected using both Alizarin Red S staining and von Kossa staining. The osteoassay surface used for bone differentiation resulted in positive staining with Alizarin Red S in the absence of any bone cells due to its calcium phosphate coating. Therefore, Alizarin Red S staining was performed on iPSCs which had been differentiated for 21 days on normal tissue culture plastic.

### Equine iPSC osteoblasts synthesise alkaline phosphatase

Alkaline phosphatase activity was detected in the undifferentiated equine iPSCs but was significantly increased by approximately sixfold over the 21 days of differentiation in OA-induction media (Fig. 2).

### The genetic background of the iPSCs affects their efficiency of differentiation into osteoblasts but there is little variation within lines derived from the same individual

The relative level of expression of osteoblast genes varied between the three lines of iPSCs that were derived from three different horses. Some lines displayed a greater level of expression of osteoblast genes than others.

**Fig. 3.**
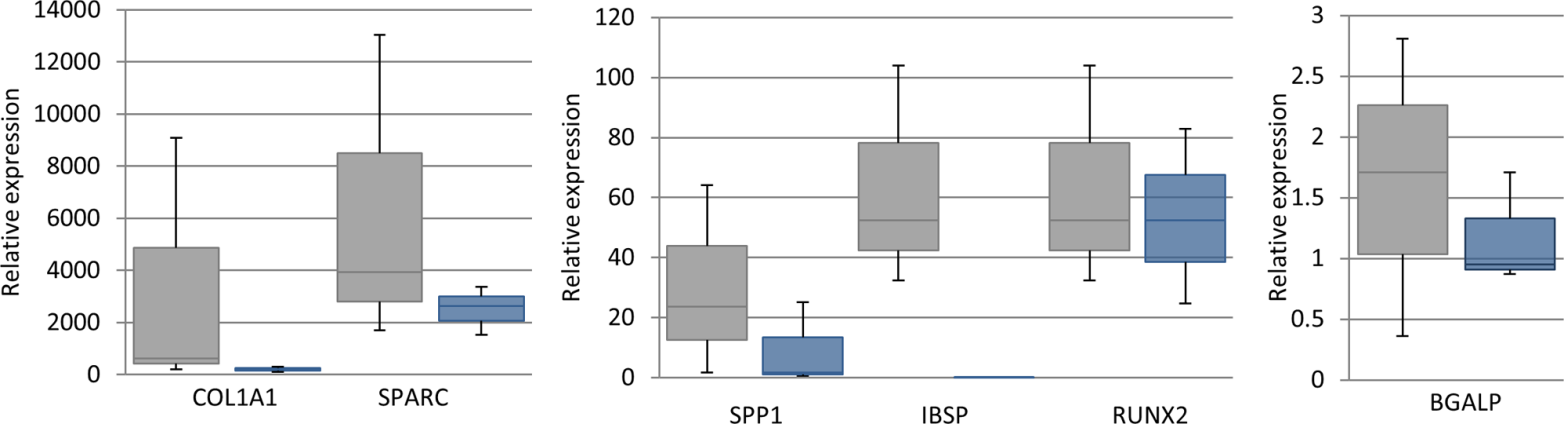
**The donor affects osteoblast gene expression by differentiated iPSCs.** Box and whisker plots to demonstrate the variation in relative osteogenic-associated gene expression as measured using quantitative PCR on three lines of iPSCs derived from different individuals (grey bars) and three lines of iPSCs derived from the same individual (blue bars). An unpaired Student's *t*-test was used to demonstrate that there are no significant differences in the mean expression value from between horses versus within one horse for any of the genes.

In contrast, three different iPSC lines isolated from the same horse were more similar in their levels of expression of osteoblast genes and, generally, had lower levels of variation than iPSC lines established from different horses (Fig. 3).

There are no significant differences in the mean expression value from between horses versus within one horse for any of the genes.

## DISCUSSION

Here, we demonstrate for the first time that equine iPSCs can differentiate directly into functional osteoblasts when cultured on an OsteoAssay surface for 21 days in osteogenic media. Equine iPSCs have been shown to differentiate into MSC-like cells that can produce mineralisation (Lepage et al., 2016), however, detailed analysis of the cells was not reported and direct differentiation from the iPSCs was not tested.

The iPSCs lines used in these experiments had either been characterised previously (Bavin et al., 2015) or they were characterised in this work for their expression of pluripotency markers and ability to undergo tri-lineage differentiation in the absence of feeder cells, LIF and bFGF (Fig. S1).

The media used in our experiments included β-glycerophosphate, ascorbic acid and dexamethasone. These factors have all been shown to be involved in driving the differentiation of stem cells into osteoblasts. β-glycerophosphate provides a source of phosphate for the formation of hydroxyapatite (HA), dexamethasone induces and enhances Runx2 activity, and ascorbic acid promotes the secretion of collagen type I (Langenbach and Handschel, 2013). We demonstrated that these factors are required for the production and deposition of calcium and hydroxyapatite by iPSCs, as 21 days of culture in non-osteogenic media did not result in detectable calcium and hydroxyapatite deposition.

A combination of both Alizarin Red S and von Kossa staining were used to detect calcium deposition. Although von Kossa is widely used to determine calcium deposition during osteoblast differentiation, it can react with the anionic portion of phosphates, carbonates, and other salts, and is therefore not specific for calcium (Bonewald et al., 2003). Alizarin Red S staining binds directly to calcium, meaning it can cross react with calcium on the surface of OsteoAssay plates. As such, Alizarin Red S staining was performed on iPSCs that were differentiated for 21 days on a normal tissue culture surface. Immunocytochemistry was performed on cells cultured on gelatin-coated coverslips rather than on OsteoAssay plates. Positive staining was observed suggesting that an OsteoAssay surface is not essential for iPSCs to differentiate into functional osteoblasts. Furthermore, we were able to detect HA deposition at similar levels on normal tissue culture and OsteoAssay plates (data not shown). Calcium phosphate coatings have previously been shown to induce the osteogenic differentiation of human iPSCs in the absence of osteogenic factors (Kang et al., 2014). In our study, we found that the culture of equine iPSCs for 21 days on the OsteoAssay plates in media lacking osteogenic factors did not result in the production of a mineralised matrix (Fig. 2). Therefore, the use of OsteoAssay plates alone was not sufficient to induce functional osteogenic differentiation of equine iPSCs in this study.

Alkaline phosphatase (ALP) is required for bone mineralisation (Golub and Boesze-Battaglia, 2007). There are four isoenzymes of ALP encoded by four genes. Three of the isoenzymes encode tissue specific ALP (intestinal, placental and germ cell), whereas the fourth isoenzyme is tissue non-specific ALP (TNAP), and different isoforms of TNAP are expressed in bone, liver and kidneys. Human and mouse ESCs and iPSCs have been reported to express TNAP (Štefková et al., 2015). Therefore, it is not surprising that we detected some ALP activity in media taken from undifferentiated equine iPSCs. However, ALP activity was significantly increased after 21 days of osteoblast differentiation. Taken together, the ALP activity and other results presented in this study support the successful differentiation of equine iPSCs into functional osteoblasts.

The expression of six genes associated with osteoblast differentiation was also determined. After 21 days of differentiation in osteoinduction media, iPSCs expressed *COL1A1*, *SPARC*, *SPP1*, *RUNX2*, *IBSP and BGALP*. *RUNX2* is a key transcription factor that drives the expression of multiple bone genes including *COL1A1*, *SPP1*, *and IBSP* (Komori, 2010). COL1A1 (collagen type I) is one of the main constituents of bone and is required for bone strength (Viguet-Carrin et al., 2006). SPARC (osteonectin) is the most abundant non-collagenous protein in bone (Termine et al., 1981). SPP1 (osteopontin) is involved in the regulation of bone remodelling and bone differentiation (Huang et al., 2004). IBSP (Integrin Binding Sialoprotein) is required for bone mineralisation and osteoblast differentiation (Bouleftour et al., 2016). BGALP (osteocalcin) is produced by mature osteoblasts and is involved in the regulation of bone mineralisation (Zoch et al., 2016). The expression of these genes by iPSCs that were cultured for 21 days in osteoinduction media supports the conclusion that these iPSCs have differentiated into osteoblasts.

We next confirmed that we could also detect proteins for all of the above osteoblast-associated markers by immunocytochemistry. We have previously demonstrated by Western blotting that the antibody to COL1A1 can cross react specifically with the equine protein (Barsby and Guest, 2013). Antibodies to SPP1 (Miragliotta et al., 2016) and SPARC (Miragliotta et al., 2009) have similarly been demonstrated to cross react specifically with their respective equine proteins. The antibodies to RUNX2 and BGALP have previously been used to detect bone differentiation in equine cells (McCarthy et al., 2012). The expression of these proteins is further proof that our iPSCs have differentiated into functional osteoblasts. Western blots of the proteins were not carried out in this study due to the technical difficulty in efficiently extracting a representative sample of protein from the mineralised matrix that had been formed by the iPSCs following their differentiation (Cleland et al., 2012).

Due to the production of a mineralised matrix it was also not possible to isolate the osteoblast-differentiated cells for flow cytometry to quantify the percentage of cells that were differentiating. Further work must therefore be performed to determine if the variability observed in the gene expression is due to lower levels of expression by osteoblast-differentiated cells, or whether it is due to a lower percentage of cells differentiating to the same degree in some lines.

Sex and genetic factors have previously been shown to influence osteoblast differentiation of mouse mesenchymal stem cells using cells derived from different strains of inbred mice (Zanotti et al., 2014). In this study, we used iPSCs derived only from male horses to avoid any variability due to sex. We demonstrated that the genetic background of the horse affects the level of osteoblast-associated gene expression after 21 days of differentiation, and large variations in the expression of osteoblast genes was observed between iPSCs from different horses. Although the donor cell type has been shown to have less effect than genetic background on the differentiation of human iPSCs (Heslop et al., 2017), donor cell type has been shown to influence the differentiation potential of equine iPSCs to other cell lineages (Quattrocelli et al., 2016). Therefore, in this study all iPSCs were derived from adult horse fibroblasts at early passage (less than P5) to ensure that any epigenetic memory of tissue source was the same between all lines (Liang and Zhang, 2013). The high levels of variation between lines derived from different animals was expected as each horse is an independent system, and the levels of expression of these genes is likely to be influenced by factors such as unique genetic polymorphisms that are maintained when iPSCs are established from each horse. The genetic background of human iPSCs has been shown to have the greatest influence on differentiation to hepatocytes (Heslop et al., 2017). However, to our knowledge this is the first time that the effect of donor variation on osteoblast differentiation from iPSCs has been examined in any species. When three clonal lines of iPSCs derived from the same horse were compared for their osteoblast-associated gene expression, we found smaller variations in the levels of expression of these genes, which is consistent with what we would expect. The inter- and intra-individual variation of *RUNX2* level was very similar, with a very similar mean value produced within and between horses. Therefore *RUNX2* was expressed at a comparable level between horses, which may reflect its importance as the master regulator of bone differentiation. The variation between lines of iPSCs derived from the same horse can be due to technical variation in the experimental set up as well as variation induced by the reprogramming process. The iPSCs used in this study were generated using integrating retroviral vectors which integrate at random sites in the genome (Varas et al., 2009). This may result in heterogeneity between clonal lines of iPSCs derived from the same individual, and the integration sites of the virus have the potential to affect the differentiation capacity of these cells (Zheng et al., 2013). All of the lines of iPSCs used in this study were generated independently to ensure that the lines derived from the same horse would have the same levels of technical variation as the lines derived from different horses. However, our results show that, irrespective of the effects that may result from sites of viral integration or any other technical variability, our iPSCs all have the capacity to differentiate into functional osteoblasts on exposure to osteoinduction media for 21 days.

Prior to their clinical application, it is necessary to derive equine iPSCs using non-integrating methods, however, to date there are no reports of this having been done in horses. Additionally, differentiation of human iPSCs into osteoblasts using 3-dimensional scaffolds has been performed (Csobonyeiova et al., 2017) and this is likely to represent the next stage of the work to move equine iPSC towards clinical applications to aid bone regeneration.

### CONCLUSIONS

In this study, we have demonstrated that equine iPSCs can be differentiated directly into functional osteoblasts. We have also shown that the genetic background of the donor affects the levels of expression of osteoblast-associated genes. This work demonstrates the future potential of using equine iPSCs as a source of cells to aid fracture repair in horses, and as a tool to study bone development and disease.

## MATERIALS AND METHODS

### iPSC derivation and culture

iPSCs were generated from male equine skin fibroblasts by retroviral transduction using methods reported previously (Bavin et al., 2015). Fibroblasts were isolated from skin biopsies of three adult male horses [taken post-mortem with the approval of the Animal Health Trust ethical review committee (AHT_02_2012)] by digesting in cell culture media [Dulbecco's modified Eagle medium (DMEM) high glucose, supplemented with 10% fetal calf serum, 1% penicillin–streptomycin, 2 mM L-glutamine and 1% fungizone (all from Invitrogen)] with 1 mg/ml collagenase type I added from *Clostridium histolyticum* (Sigma-Aldrich) and incubated at 37°C overnight. Fibroblasts were cultured in this media but without fungizone at 37°C, 5% CO_2_ until confluent. Fibroblast cells were passaged at confluency with trypsin-EDTA (Sigma-Aldrich) for expansion and stocks were frozen in media with 10% DMSO. iPSC generation was performed as described in Baird et al. (2015) and Bavin et al. (2015). Briefly, phoenix gag-pol packaging cells were transfected with 3 µg of pVPack-VSV-G (Agilent technologies, Stockport, UK) along with 3 µg of pMXs.hOct4 (Addgene 17217), pMXs.hSox2 (Addgene 17218), pMXs.hKlf4 (Addgene 17219), pMXs.hc-Myc (Addgene 17220), or pMX.GFP (Cell Biolabs, San Diego, USA). Transfections were carried out using lipofectamine 2000 and Opti-MEM media (both Invitrogen) according to the manufacturer's instructions. After 48 h culture supernatant containing the viral particles was pooled, filtered through a 0.45 µM filter (Nalgene), supplemented with 1 µg/ml polybrene (Sigma-Aldrich) and used to infect equine skin fibroblasts which had been plated at a density of 1×10^4^ the day before infection.

Three rounds of viral infection were carried out at 48 h intervals prior to plating the infected cells at a density of 5×10^3^ cells per 10 cm plate pre-seeded with feeder cells (mitotically inactivated mouse embryonic fibroblasts). The media was replaced with iPSC media [DMEM/F12, supplemented with: 15% FCS, 2 mM L-glutamine, 1% non-essential amino acids, 1 mM sodium pyruvate, 0.1 mM 2-mercaptoethanol (all Invitrogen), 1000 U/ml leukaemia inhibitory factor (LIF, Sigma-Aldrich), and 10 ng/ml basic fibroblast growth factor (bFGF, Peprotech, London, UK)]. iPSC media was replaced every other day until iPSC colonies began to appear and reached a large enough size to manually pick selected colonies. These colonies were used to establish clonal iPSC lines. iPSCs were mechanically passaged in the presence of 2 μM Thiazovivin (StemGent, Cambridge, USA) and frozen in iPSC media containing 10% DMSO. iPSCs were used at passage 9–15 for all osteoblast differentiation studies. For spontaneous differentiation to confirm pluripotency, the iPSCs were cultured in iPSC media without LIF, bFGF or feeders for 7 days prior to use in immunocytochemistry. For embryoid body formation the iPSCs were plated in this media on low attachment plates (Corning Life Sciences, Tewksbury, USA) for 14 days.

### Osteoblast differentiation

To induce osteoblast differentiation, iPSCs were plated (in colony pieces) at a density of 7×10^4^ cells per well of a 24-well OsteoAssay surface coated plate (Corning, Wiesbaden, Germany) in iPSC media. The following day, the media was replaced with osteogenic media [DMEM/F12, supplemented with: 15% FCS, 2 mM L-glutamine, 1% non-essential amino acids, 1 mM sodium pyruvate, 0.1 mM 2-mercaptoethanol (all Invitrogen), 10 mM β-glycerophosphate, 10 nM dexamethasone and 28 µM ascorbic acid (all Sigma-Aldrich)]. Cells were cultured for 21 days with the media replaced every 2–3 days prior to analysis.

To determine matrix mineralisation, von Kossa staining (Abcam, Cambridge, UK) was carried out according to the manufacturer's instructions. Alizarin Red S staining for calcium deposition was also performed by incubating differentiated iPSCs with 2% Alizarin Red S pH 4.2 for 5 min. Hydroxyapatite deposition was detected using the OsteoImage bone mineralisation assay (Lonza, Slough, UK) according to the manufacturer's instructions. Alkaline phosphatase activity was measured using a quantitative colorimetric test on cell culture supernatant (Abcam) according to the manufacturer's instructions.

### RNA extraction, CDNA synthesis and quantitative PCR

RNA was extracted using Tri-reagent (Sigma-Aldrich), purified using the RNeasy mini kit (Qiagen) and treated with Ambion DNA-free (Life Technologies). cDNA was made from 1 μg of RNA using the sensiFAST cDNA synthesis kit (Bioline, London, UK). 2 μl aliquots of cDNA were used in qPCR. Primers were designed using NCBI Primer-Blast (https://www.ncbi.nlm.nih.gov/tools/primer-blast/). Primer sequences can be found in Table 1. Quantitative PCR (qPCR) was carried out using SYBR Green containing supermix (Bioline) on the Bio-Rad C1000 Touch Thermal Cycler (Bio-Rad), and all PCR reactions performed in duplicate. PCR cycle parameters were 95°C for 10 min, followed by 40 cycles of 95°C for 15 s, 60°C for 15 s and 72°C for 15 s. At the end of the program a melt curve was produced by taking readings every 1°C from 65°C to 95°C. The levels of 18S rRNA did not change between treatments (data not shown) and it was used to normalise gene expression using the 2^−ΔΔCt^ method (Livak and Schmittgen, 2001). An unpaired Student's *t*-test was used to determine significant differences in the mean gene expression using iPSCs from three independent horses versus three lines of iPSCs derived from the same horse.

**Table 1.**
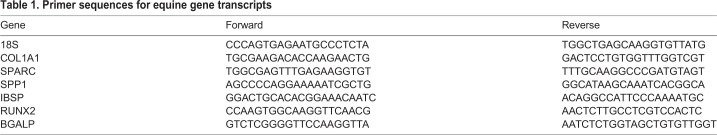
**Primer sequences for equine gene transcripts**

### Immunocytochemistry

Cells were cultured on gelatin-coated (Sigma-Aldrich) coverslips, fixed in 3% paraformaldehyde for 20 min and permeabilised for 1 h with 0.1% triton-X-100. They were washed in PBS and incubated with the primary antibodies overnight at 4°C before detection with an appropriate fluorescently labelled secondary antibody. All antibodies were used at optimized concentrations in PBS and appropriate negative controls were performed using secondary antibodies alone and IgG matched to the host species, as well as specific isotype of the primary antibody. Coverslips were mounted using Vectashield Hardset mounting medium containing DAPI (4′,6-diamidino-2-phenylindole, Vector Laboratories, Cambridge, UK). Primary antibodies included: rabbit anti-alpha fetoprotein 1:500 (Biorbyt, orb7822, Cambridge, UK), mouse anti-actin 1:200 (Dako, M0635, Cambridge, UK), mouse anti-Beta-III tubulin 1:100 (Sigma-Aldrich, T8660), mouse anti-SSEA-4 1:100 (MAB4304, Millipore), mouse anti-SSEA-1 1:100 (Chemicon, MAB4301), rat anti-SSEA-3 1:100 (Chemicon, MAB4303), mouse anti-TRA-1-60 1:500 and mouse anti-TRA-1-81 1:500 (both kindly provided by Prof Peter Andrews at the University of Sheffield, UK), rabbit anti-Oct 4 1:100 (Abcam, Ab18976), mouse anti-collagen type I 1:100 (Abcam, Ab90395), mouse anti-osteonectin (SPARC) 1:20 (Bio-Techne, mab941-100, Oxford, UK), mouse anti-osteopontin (SPP1) 1:50 (Santa Cruz Biotechnology, sc21742), rabbit anti-bone sialoprotein (IBSP) 1:100 (Biorbyt, orb1100, Cambridge, UK), rabbit anti-Runx2 1:50 (Santa Cruz Biotechnology, sc10758), goat anti-osteocalcin (BGALP) 1:50 (Santa Cruz Biotechnology, sc18319). Secondary antibodies were goat anti-mouse alexafluor 594 1:200 (Invitrogen, A11005), goat anti-rabbit alexafluor 594 1:100 (Invitrogen, A11012), donkey anti-goat FITC (Abcam, ab7121) and goat anti-rat Texas Red 1:200 (Sigma-Aldrich, SAB3700668).

## Supplementary Material

Supplementary information
