# Supplementary material for: Osteoblast differentiation of equine induced pluripotent stem cells
Source: Biol Open. 2018 Apr 23;7(5):bio033514. doi: 10.1242/bio.033514 (PMC5992527; doi:10.1242/bio.033514)
Supplement: Supplementary information [file biolopen-7-033514-s1.pdf]

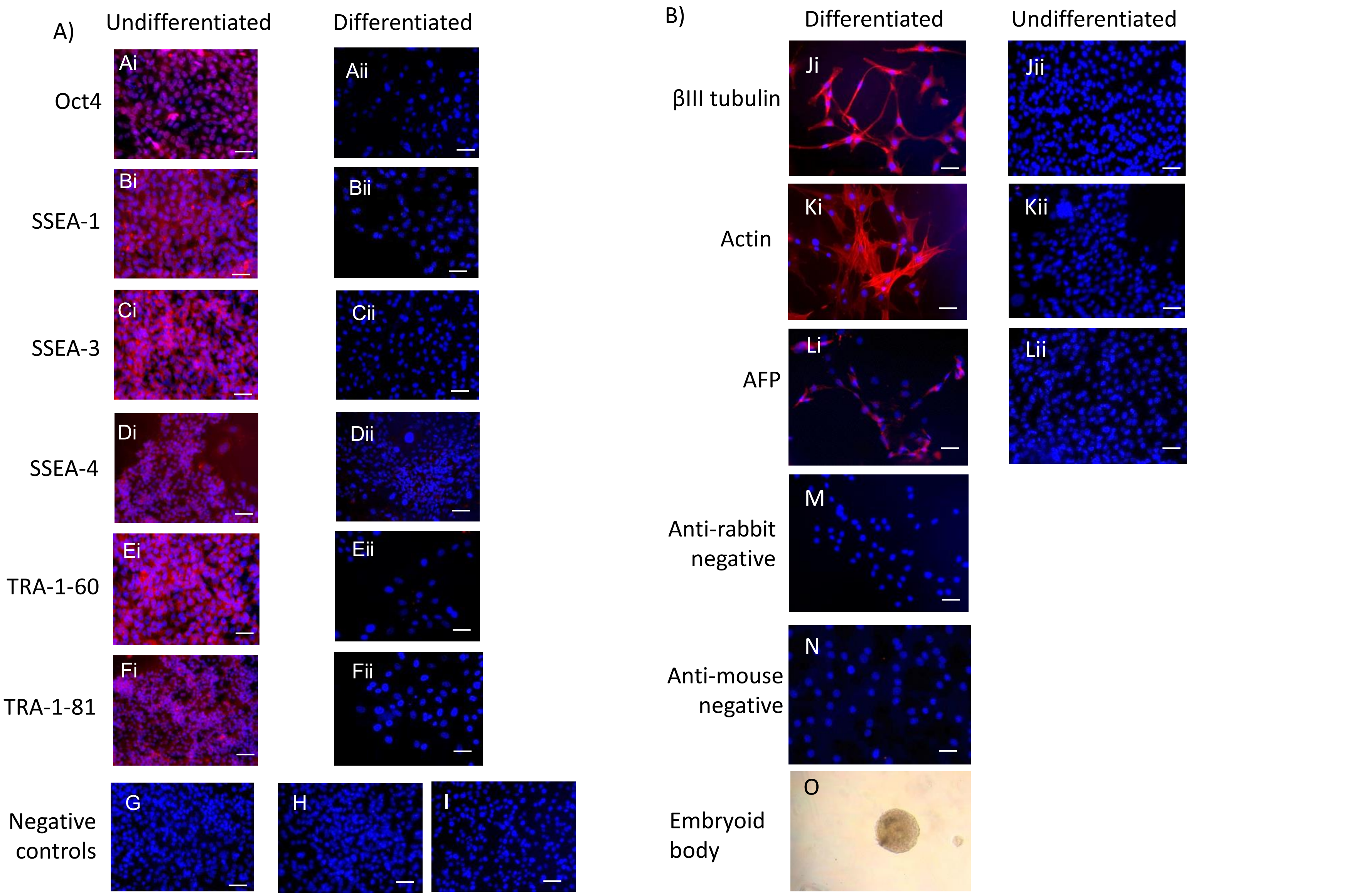

Supplementary Figure 1A). Undifferentiated iPSCs express pluripotency markers (Ai – Fi) and these are lost upon spontaneous differentiation (Aii – Fii). Negative controls on the undifferentiated cells for the secondary antibodies are shown in G) anti-mouse, H) anti-rabbit and I) anti-rat. **B)** iPSCs can undergo differentiation *in vitro*. After 7 days of spontaneous *in vitro* the iPSCs exhibit positive staining for βIII tubulin, actin and alpha-fetoprotein (Ji-Li). These proteins are not produced by the undifferentiated iPSCs (Jii-Lii). Negative controls on the differentiated cells are shown in M and N. Scale bar = 40 μm. The iPSCs can also form embryoid bodies in suspension culture over a period of 14 days (O).
